# Effects of Fishmeal Substitution with Mealworm Meals (*Tenebrio molitor* and *Alphitobius diaperinus*) on the Growth, Physiobiochemical Response, Digesta Microbiome, and Immune Genes Expression of Atlantic Salmon (*Salmo salar*)

**DOI:** 10.1155/2024/6618117

**Published:** 2024-01-06

**Authors:** H-Michael Habte-Tsion, Matt Hawkyard, Wendy M. Sealey, David Bradshaw, Kala-Mallik Meesala, Deborah A. Bouchard

**Affiliations:** ^1^Aquaculture Research Institute and Cooperative Extension, University of Maine, Orono, ME 04469, USA; ^2^Bozeman Fish Technology Center, USDA—ARS, Bozeman, MT 59715, USA; ^3^Department of Aquaculture and Stock Enhancements, Harbor Branch Oceanographic Institute, Florida Atlantic University, Fort Pierce, FL 34946, USA

## Abstract

A 12-week growth trial was conducted to assess the effects of mealworm meals, as a substitution for fishmeal, on the growth, physiobiochemical responses, digesta microbiome, and immune-related genes expression of Atlantic salmon (*Salmo salar*). Twenty Atlantic salmon parr (38.5 ± 0.1 g, initial weight) were stocked into each of 16 tanks in a recirculating aquaculture system. A fishmeal-based diet (100% FM) was used as the control treatment and was compared with three test diets where: (1) fishmeal was partially (50%) replaced with defatted mealworm meal, *Tenebrio molitor* (50% DMM), (2) fishmeal was fully replaced with defatted mealworm meal (100% DMM), and (3) fishmeal was partially replaced with whole lesser mealworm meal, *Alphitobius diaperinus* (50% WMM). All substitutions were done on a crude protein basis. Each of the four experimental diets was evaluated in quadruplicate tanks as part of randomized design. The results indicated that Atlantic salmon showed high survival (greater or equal to 98.8%), and no significant difference in final growth, feed efficiency, feces stability and condition indices. Hepatosomatic index was lower in fish fed 100% DMM and 50% WMM when compared to fish fed the control diet (100% FM). Whole-body proximate and amino acid compositions were not statistically different between treatments, while essential fatty acids, including linolenic, eicosapentaenoic acid, and homo-a-linolenic, were lower in fish fed 100% DMM. Plasma parameters (total protein, alanine aminotransferase, alkaline phosphatase, and total iron-binding capacity), hepatic peroxide, and antioxidant enzymes were not significantly affected by dietary substitutions, whereas plasma immunoglobulin M showed significantly higher levels in fish fed 50% DMM and 100% DMM when compared to fish fed the control diet (100% FM). The inclusion of mealworm meals significantly impacted the overall microbiome composition but not the richness and evenness of the salmon digesta microbiomes compared to control. The most common genus in all treatments was *Pseudomonas*, which has been previously shown to have both commensal and pathogenic members. The relative expressions of growth (IGF-I) and protein synthesis (TIPRL) were not significantly different between the treatments, whereas immunoglobulin genes (IgM, IgD, and IgT) were significantly upregulated in fish fed the DMM diets when compared to fish fed the control diet. Overall, this study suggests that the mealworm meals tested could be suitable alternatives to fishmeal in the diet of Atlantic salmon.

## 1. Introduction

Aquaculture is one of the fastest growing food production industries globally, and finfish farming has accounted for the largest share of world aquaculture for decades. In 2020, farmed finfish reached 57.5 million tons and in particular, farmed Atlantic salmon has been one of the major contributors to growth in the global aquaculture production and global trade [1]. However, there are increasing challenges including environmental impact and cost of aquafeeds ranged 50%–60% of the operational costs. Currently, fishmeal is the primary ingredient used as a quality protein source in feeds for aquatic animals, particularly those used for salmon and other carnivorous fish species. Nevertheless, the utilization of fishmeal as the primary source of protein in aquafeeds is becoming unfeasible, both practically and economically, due to its limited supply and rapidly increasing price. Therefore, developing new alternative ingredients, including those derived from insects, to replace fishmeal is a priority for the aquaculture industry and scientists.

Insect meals are among the most promising novel protein sources in aquafeeds due to their nutritional compositions such as protein content and amino acid profile as well as their potential for commercial-scale production to meet the demand for alternative protein sources in aquafeeds [2–7]. Mealworms are insects rich in proteins (47%–60% on a dry weight basis; DW) and lipids (31%–43% DW) and with favorable amino acid and fatty acid profiles for animal feeds [8]. There are increasing reports of partial to complete replacement of fishmeal by insect meals, including mealworm meals in the diets of carnivorous finfish such as rainbow trout, *Oncorhynchus mykiss* [9–12], European sea bass, *Dicentrarchus labrax* [13, 14], gilthead seabream, *Sparus aurata* [15], and other finfish species such as African catfish, *Clarias gariepinus* [16, 17], common catfish, *Ameiurus melas* [18], yellow catfish, *Pelteobagrus fulvidraco* [19], and Tilapia, *Oreochromis nilotica* [20]. Moreover, insects, including mealworms, contain varying amounts of chitin [21] which may have immunomodulatory benefits in aquatic animals [14, 22]. Few studies have reported the effects of mealworm meals on the immune system of fish, which mainly focus to European sea bass [14], Jian carp, *Cyprinus carpio* var. Jian [23], and yellow catfish [19].

In fish nutrition studies, blood biochemistry and hepatic antioxidants are key parameters for evaluating fish health as it relates to nutritional manipulations [14, 24–32]. The adaptive immune system of teleosts includes B cells, which produce three classes of immunoglobulins (Igs) such as IgM, IgD, and IgT (or IgZ in some species), with IgM+ being the predominant surface Ig isotype [33, 34]. The relative expression of target genes related to immune/health responses as a result of dietary substitutions is important because they can provide insights that show further specific mechanisms behind fish health [19, 23, 35, 36]. The gut microbiome includes both allochthonous (transient) members that are more associated with the dietary components found in the digesta in the lumen of the gut which is eventually excreted in the feces as well as autochthonous (residential) members which are associated with the host's gut epithelial surface or microvilli [37]. The gut microbiome changes due to diet, exposure to toxicants, stress, and other environmental factors such as salinity and temperature [38]. These changes in gut microbiome can affect the nutritional metabolism, immune regulation, fish development, and disease resistance of the fish host [39]. Studies have shown that the use of mealworm meal has had variable effects on the gut microbiome of rainbow trout, gilthead sea bream, and European sea bass [40, 41].

Furthermore, recent growth in land-based recirculating aquaculture systems (RASs) has created the need to develop diets suitable for use in closed systems. In RAS, feeds that result in physically stable feces are desired to minimize the disintegration of these waste products into suspended solids which can degrade water quality and may compromise fish health. In general, larger particle sizes (>150 microns) of suspended solids are desired as more efficiently removed by mechanical filtration [42]. Feces stability is believed to be affected by the feed ingredients used because they may contain natural surfactants or other compounds that relate to particle binding or breakdown. This study hypothesis that diets produced with mealworm meals are suitable for use in RAS, particularly in terms of the resultant feces' stability and larger particle sizes of suspended solids, more efficiently removable by filters.

This study was conducted to determine if the substitution of fishmeal with two mealworm meals in the diet of Atlantic salmon effected the growth performance, feed utilization, feces stability, condition indices, body composition, health parameters, digesta microbiome, and the relative expression of target genes related to fish growth, protein synthesis, and health.

## 2. Materials and Methods

### 2.1. Experimental Diets

Table 1 shows the nutritional values of the test ingredients used in this study which included defatted mealworm meal, *Tenebrio molitor*, and whole mealworm meal, *Alphitobius diaperinus* (Ÿnsect, France). The formulations of the control diet (100% FM) produced with a high-quality fishmeal (FM) with 75% crude protein (SeaProTM 75, BioOregon Protein Inc., Warrenton, OR, USA) as a primary ingredient and three test diets (partial and full substitution of FM by defatted yellow mealworm meal (50% DMM and 100% DMM, respectively) and partial replacement of FM with whole mealworm meal (50% WMM)) are presented in Table 2. Complete replacement of fishmeal with whole mealworm meal could not be achieved because the high lipid concentrations of WMM could not be balanced using practical feed formulations. All ingredient replacements were conducted on a crude protein basis. The four diets were formulated to be isonitrogenous, isolipidic, and isoenergetic while considering similar levels of total fiber and meeting the requirements of Atlantic salmon for essential amino acids and phosphorus. Guar gum (0.3% w/w on a dry weight basis) was added as a binding agent known to stabilize salmonid feces [43]. Diets were produced at the USDA-ARS facility in Bozeman, MT, USA, using a twin-screw extruder (DNDL-44, Buhler AG, Uzwil, Switzerland). Pellets were then dried, cooled, and vacuum coated with oil blends. Analyses of experimental diets for proximate, amino acid, and fatty acid compositions (Tables 3 and 4) were carried out by the Experiment Station Chemical Laboratories (ESCL), University of Missouri, Columbia, MO, USA.

### 2.2. Experimental System and Approach

A 12-week growth trial was conducted to evaluate the four diets described above. Each diet was evaluated in four replicates using a randomized design. The use of experimental fish was under scientific research protocols of the University of Maine Institutional Animal Care and Use Committee (Protocol number A2021-09-04) and complied with all relevant international animal welfare laws, guidelines, and policies. Twenty feed-trained Atlantic salmon parr (38.5 ± 0.1 g initial weight) were stocked into each of the 16 experimental tanks of the RAS system, with tank serving as the experimental replicate. Diets were randomly assigned to tanks using a random number generator (random.org). Following stocking and acclimation, experimental diets were fed to Atlantic salmon three times daily to apparent satiation/ ad-libitum (09:00, 13:00, and 16:00 hr) and two times daily (09:00 and 16:00 hr) during weekends for 12 weeks. Feed given per tank was recorded daily by weighing feed before and after feeding time. Fish were weighed three times: at the beginning, middle, and end of the growth trial. Water quality parameters were maintained within suitable ranges for Atlantic salmon, including temperature (12–14°C), pH (7–8), dissolved oxygen (80%–120%), total ammonia nitrogen (0–0.8 mg/L), and nitrite nitrogen (0–0.6 mg/L). Photoperiod was 12-hr daylight and 12-hr darkness (artificial lighting controlled by a timer).

### 2.3. Sample Collection

At the beginning of the growth trial, samples of diets and 10 fish (∼40 g each) from the source population were collected and stored at −80°C for proximate, amino acid, and fatty acid composition analyses. At the end of the 12-week growth trial, fish in each tank were sampled after a 24-hr fasting period. All fish from each tank were bulk weighed and counted. Five fish were returned to their respective tanks and cultured for an additional 1 week at which point they were sampled for digesta microbiome analysis and evaluation of feces stability. Three fish from each experimental tank were bled following anesthesia with buffered tricaine methanesulfonate (MS-222; buffered with 200 mg/L of sodium bicarbonate) at approximately 75 mg/L. Blood samples were placed into 2 mL tubes, with lithium heparin (Greiner Bio-One North America Inc., Monroe, NC, USA) as anticoagulant. Plasma was extracted immediately by microcentrifugation at 3,000x *g* at 4°C for 10 min. Three additional fish were sampled and humanely euthanized with 250 mg/L buffered MS-222. The external surface of each euthanized fish was wiped with 70% ethanol to avoid contamination from external microbes, and liver was removed and placed into a 2 mL microcentrifuge tube and stored at −80°C for analysis of hepatic peroxide and antioxidant enzymes. Next, part of the midgut of the same three fish per tank was removed and placed in a separate group of sterile 2 mL tubes and stored at −80°C for conducting growth and immune-related gene expression assays. Eleven euthanized fish from each tank were individually weighed and measured to calculate the Fulton condition factor (K-factor); and then five fish were dissected, and their viscera were used to calculate viscerosomatic index (VSI). Six euthanized fish from each tank were stored at −80°C for subsequent whole-body proximate, amino acid, and fatty acid composition analyses.

The remaining five fish per tank were fed with their respective experimental diets for one additional week and then euthanized, as described above. The digestive tracts of these fish were aseptically dissected and the digesta was removed by massaging the GI until the digesta pellet emerged and frozen at −80°C for microbiome analysis (two fish per tank and eight fish per treatment) and evaluation of feces stability (three fish per tank and 12 fish per treatment).

### 2.4. Feces Stability Assay

Fecal strands were thawed 2–3 hr prior to analysis. Approximately 50 mg (wet weight) of undisturbed fecal material was added to a laser diffraction particle size analyzer (Mastersizer 3000, Malvern Panalytical, Malvern, UK) equipped with a Hydro MV automated dispersion unit (Malvern Panalytical, Malvern, UK). Feces breakdown was achieved by stirring (800 rpm) each sample in distilled water, using the Hydro MV stirrer, for 20 s prior to the first measurement. The particle size distribution (percent by total particle volume) of each sample was measured five times and then averaged. The mean particle size at the 10^th^, 50^th^, and 90^th^ percentiles of the resultant particle size distributions was used in statistical comparisons.

### 2.5. Chemical Composition Analysis

Test ingredients, feed, and whole-body samples were analyzed for total crude protein, energy, total (ether extracted) crude lipid, moisture, amino acid, and fatty acid compositions. The proximate composition of diets and whole-body samples was analyzed using standard procedures [44]. The amino acids and fatty acids analyses were conducted by the Experiment Station Chemical Laboratories (ESCL), University of Missouri, Columbia, MO, USA, using methods approved by the Association of Official Analytical Chemists (AOAC) and American Oil Chemists' Society (AOCS).

### 2.6. Plasma Biochemical, Hepatic Peroxide, and Antioxidants Analyses

Plasma biochemistry (alanine aminotransferase (ALT), alkaline phosphatase (ALP), total protein (TP), immunoglobulin M (IgM), and total iron-binding capacity (TIBC)) and hepatic peroxide (malondialdehyde (MDA)) content, antioxidants enzymes (superoxide dismutase (SOD) and glutathione peroxidase (GPx)) activity, were analyzed spectrophotometrically using commercial kits (BioVision, Milpitas, CA, USA), as described in previous studies [31, 32, 35, 45].

### 2.7. Extraction, Sequencing, and Sequence Analysis for Digesta Microbiome

Triplicate samples of each of the feeds (*n* = 12) as well as duplicate digesta samples (*n* = 32) from each tank were sent to RTL Genomics (Lubbock, TX, USA) for 16S metabarcoding. Once received by RTL Genomics, the digesta and feed samples underwent DNA extraction with the Zymo ZR-96 Magbead kit (Irvine, CA, USA) on the Thermo Scientific KingFisher FLEX instrument (Waltham, MA, USA) using a modified version of the manufacturer's instructions which included a mechanical lysis step with a Qiagen TissueLyser (Hilden, Germany). Samples were amplified using the 28F (GAGTTTGATCNTGGCTCAG)-519R (GTNTTACNGCGGCKGCTG) PCR primers to amplify the V1–V3 region of the 16S gene [46]. The amplicons were sequenced on an Illumina MiSeq (San Diego, CA, USA).

Demultiplexed raw sequences were analyzed with two Snakemake protocols: one focused on quality control and trimming using Trim Galore and FastX, respectively, and another based around QIIME2 (v2022.2) [47–51]. The second Snakemake used Dada2 to denoise the sequences and Vsearch to annotate the resulting amplicon sequence variants (ASVs) with an RESCRIPT-built version of the 138.1 SILVA database focusing on the V1–V3 region of 16S [52–57]. ASVs associated with mitochondria, chloroplast, eukaryotes, and unassigned annotations were removed. See *Supplementary 1* for further information regarding changes in number of sequences per sample through analysis.

### 2.8. RNA Isolation, Reverse Transcription, and Real-Time qPCR Assays

RNA isolation, reverse transcription, and real-time qPCR assays for target genes were conducted, according to previous studies [31, 35, 58]. Total RNA was isolated from the intestine of Atlantic salmon using TRIzol® reagent (Life Technologies, Carlsbad, CA, USA). A NanoDrop™ spectrophotometer (Thermo Scientific™, USA) was used to assess quality and quantity of the isolated RNA. The cDNA of samples was synthesized using PrimeScript™ RT reagent kit (Takara Bio, San Jose, CA, USA) following the manufacturer's instructions. Specific primers for target genes related to growth (insulin-like growth factor-I (IGF-I)), protein synthesis (target of rapamycin signaling pathway regulator-like (TIPRL)), and immunoglobulins (IgM, IgD, and IgT) were designed using online resources according to the partial cDNA sequences of the target genes using Atlantic salmon transcriptome analysis based on published sequences (Table 5). All primers of the target and housekeeping genes were synthesized by the Integrated DNA Technologies (IDT, Morrisville, NC, USA).

Real-time qPCR was used to determine mRNA levels for the target genes and was performed according to standard protocols provided by the manufacturer. The real-time qPCR was carried out in a RT-qPCR System (QuantStudio3, Applied Biosystems). The thermocycling conditions for the target genes were initiated with the denaturation step at 95°C for 30 s followed by 40 cycles at 95°C for 5 s, 60°C for 34 s, and 95°C for 30 s, 95°C for 3 s, 60°C for 30 s, respectively. Melting curve analysis was performed to verify that a single PCR product was produced. Threshold cycle number (*C*_T_) for each sample was determined using the software provided with the qPCR system, which was related to the concentration of the target genes. A housekeeping gene of Atlantic salmon (*β*-actin) was used to normalize the expression levels of the target genes. The amplification efficiencies of the target and housekeeping genes were quantified according to the specific gene standard curves generated from 10-fold serial dilutions. After verifying that the primers were amplified with 100% efficiency, the relative expression results were analyzed using the 2^−*ΔΔ*Ct^ method [60].

### 2.9. Calculation

The following performance parameters were used to assess the response of the experimental fish to the various dietary treatments:

(1) $$\begin{array}{c} \text{Survival }\left(\%\right)=\left(\text{Final population}/\text{initial population}\right)\times 100. \end{array}$$

(2) $$\begin{array}{c} \text{Weight gain }\left(\%\right)=\left(\left(\text{Final body weight}-\text{initial body weight}\right)/\left(\text{initial body weight}\right)\right)\times 100. \end{array}$$

(3) $$\begin{array}{c} \text{Feeding rate},\text{ percentage of body weight per day }\left(\text{BW }{\text{day}}^{-1}\right)=\left(\text{Dry feed intake }\left(g\right)/{\left(\text{initial body weight}\times \text{final body weight }\left(g\right)\right)}^{1/2}/\text{days on feed}\right)\times 100. \end{array}$$

(4) $$\begin{array}{c} \text{Feed efficiency }\left(\text{FE}\right)=\text{Weight gain }\left(g\right)/\text{dry feed consumed }\left(g\right). \end{array}$$

(5) $$\begin{array}{c} \text{Protein efficiency ratio }\left(\text{PER},\;\%\right)=\left(\text{Weight gain }\left(g,\text{ wet weight}\right)/\text{protein intake }\left(g,\text{ dry weight}\right)\right)\times 100. \end{array}$$

(6) $$\begin{array}{c} \text{Fulton condition factor},\;K-\text{ factor }\left(\%\right)=\left(\text{Fish weight }\left(g\right)/{\left(\text{fish length},\text{ cm}\right)}^{3}\right)\times 100. \end{array}$$

(7) $$\begin{array}{c} \text{Hepatosomatic index }\left(\text{HSI},\;\%\right)=\left(\text{Liver weight }\left(g\right)/\text{body weight }\left(g\right)\right)\times 100. \end{array}$$

(8) $$\begin{array}{c} \text{Viscerosomatic index }\left(\text{VSI},\;\%\right)=\left(\text{Viscera weight }\left(g\right)/\text{body weight }\left(g\right)\right)\times 100. \end{array}$$

### 2.10. Statistical Analysis

Growth performance, feces stability, body composition, physiobiochemical, and nutrigenomics data were validated for normality and homogeneity of variances by Shapiro–Wilk and Levene's tests, respectively. Thereafter, these data were analyzed using SPSS version 27 (SPSS, Chicago, IL, USA) and subjected to one-way analysis of variances (ANOVA) followed by Tukey's HSD test. All results were considered significantly different at the level of *P* < 0.05.

The final microbiome abundance table was imported into RStudio (version 2022.07.2, R version 4.2.0) with the following libraries loaded into the environment: phyloseq (1.42.0), vegan (2.6-4), ggplot2 (3.4.0), plyr (1.8.8), tidyverse (1.3.2), dplyr (1.0.10), reshape (0.8.9), DESeq2 (1.38.2), rstatix (0.7.1), and ANCOMBC (2.0.1) [61–66]. Sequences were filtered of low abundance ASVs (<11 sequences) to reduce the possible noise they could introduce to patten detection at a community scale [67]. The untransformed ASV counts were used to calculate the alpha diversity which was represented in this study by Shannon diversity. Shannon diversity was statistically correlated with observed ASVs and Simpson diversity (Spearman Rank Correlation Benjamini Hochberg adjusted *P*-values < 0.05). Since the Shannon diversity was not normal (Shapiro–Wilk test *P*-value = 2.10E−7) and heteroscedastic (Bartlett test = 0.00069), the Welch's analysis of variance (ANOVA) and Games–Howell tests (with *P*-value adjusted using Tukey's method) were used to determine statistical significance between diets. To assess the beta diversity, abundances were variance-stabilizing transformed with DESeq2 before using Bray–Curtis to create a dissimilarity matrix which was imported to PRIMER7/PERMANOVA+ to calculate overall and pairwise permutational analysis of variance (PERMANOVA) between diets with 9,999 permutations, the unrestricted method, Type III Sum of Squares, and Monte Carlo adjusted *P*-values [68]. Differences between samples were visualized with a principal coordinate of analysis (PCoA). Differential abundance between the control diet (100% FM) and each of the three mealworm meal diets (50% DMM, 100% DMM, and 50% WMM) were determined with ANCOMBC2 (Analysis of Composition of Microbiomes with Bias Correction 2) with *P*-values adjusted with the default Holm method. The Snakemake, Conda environment, R scripts, and R session files can be found on GitHub (https://github.com/djbradshaw2/Atlantic_salmon_mealworm_meal). Sequences were uploaded into the NCBI database (PRNJ accession number: PRJNA916945).

## 3. Results

### 3.1. Growth Performance, Feed Utilization, and Feces Stability

**Figure 1:**
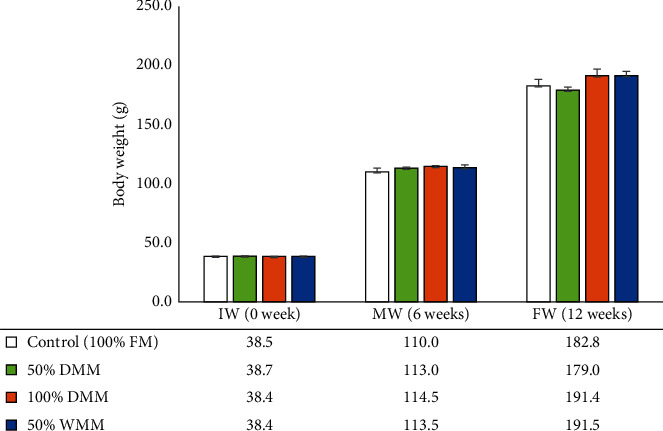
Effects of FM substitution by mealworm meals (at 50% DMM, 100% DMM, and 50% WMM) on the final weight at different weighing periods (initial, middle, and final) of Atlantic salmon fed the experimental diets for 12 weeks. Values are means with standard errors represented by vertical bars (*n* = 4). FM, fishmeal; DMM, defatted mealworm meal; WMM, whole mealworm meal; IW, initial weight; MW, middle weight; and FW, final weight.

Growth performance, survival, feed utilization, and condition indices of Atlantic salmon fed the experimental diets for 12 weeks are presented in Table 6. Survival of Atlantic salmon at 12 weeks of feeding trial was 100% in all treatments with the exception of the 100% DMM treatment which lost one fish and had a survival of 98.8% (ANOVA, *P*=0.426). Feeding rates were not significantly different among treatments (ANOVA, *P*=0.133). No significant differences were observed in the weight gain, feeding efficiency (FE) ratio, protein efficiency ratio, Fulton's condition factor (K-factor), and VSI. Fish fed with 100% DMM and 50% WMM displayed significantly lower hepatosomatic index than fish fed the control diet (100% FM; *P* < 0.05). In addition, the substitution of FM with DMM and WMM did not significantly affect the final weight of Atlantic salmon at the middle and final weighing periods (Figure 1).

**Figure 2:**
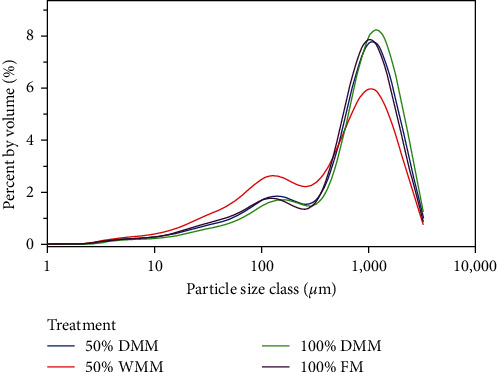
Histogram showing particle size distribution (log scale; % volume) of feces breakdown products after physical agitation for 20 s. Treatments are represented by different colors: blue = 50% DMM, red =  50% WMM, green = 100% DMM, and purple = 100% FM.

Feces stability: particle size distributions as indicated by the mean particle size at the 10^th^, 50^th^, and 90^th^ percentiles were not significantly different between treatments (ANOVA, *P*=0.096, 0.124, and 0.167, respectively). The resultant histograms are shown in Figure 2.

### 3.2. Whole-Body Proximate, Amino Acids and Fatty Acids Composition

**Figure 3:**
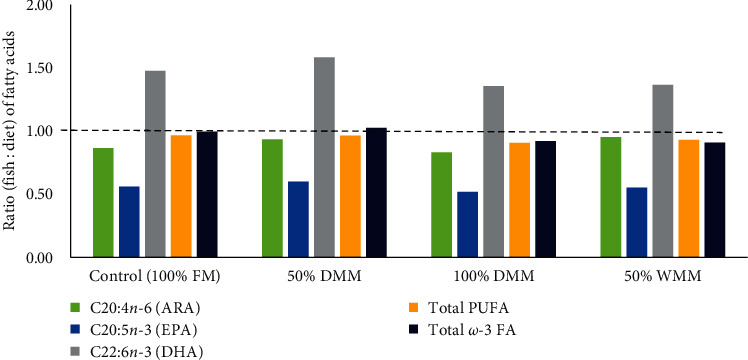
Ratio (fish : diet) of fatty acids in Atlantic salmon (*Salmo salar*) parr after a 12-week feeding trial with a control diet (100% FM), and three test diets such as 50% DMM, 100% DMM, and 50% WMM. The dashed line (ratio = 1) indicates equal amounts of fatty acids in fish and the diet. DMM, defatted mealworm meal; WMM, whole mealworm meal; ARA, arachidonic acid; EPA, eicosapentaenoic acid; DHA, docosahexaenoic acid; total PUFA, total polyunsaturated fatty acids; and total *ω*-3 FA, total omega-3 fatty acids.

Table 7 presents the whole-body proximate and amino acid compositions of Atlantic salmon fed the experimental diets for 12 weeks. There were no significant differences in the proximate and amino acid compositions between the treatments (ANOVA, *P* > 0.05 for all indices). Whole-body fatty acid compositions of Atlantic salmon fed the experimental diets for 12 weeks are reported in Table 8. The results showed that fatty acids (including EPA, linolenic, clupanodonic, homo-*α*-linolenic) were significantly influenced by the dietary substitutions (ANOVA, *P*=0.025, 0.001, 0.031, and 0.010, respectively) with lower values in fish fed 100% DMM. However, DHA (one of the omega-3 fatty acids) content in the whole body of Atlantic salmon was not significantly different between treatments (ANOVA, *P*=0.051). In addition, the ratios of fish to diet (FD ratio) for ARA, EPA, total PUFA, and total omega-3 fatty acids were similar in all treatments and were ≤1.0, while the FD ratio for DHA was >1.0 in all groups (Figure 3).

### 3.3. Plasma and Liver Biochemical Parameters

Table 9 shows plasma health parameters, hepatic peroxides (MDA), and antioxidants of Atlantic salmon fed the experimental diets for 12 weeks. Plasma ALT, ALP, TP, and TIBC were not significantly different among treatments (ANOVA, *P*=0.063, 0.557, 0.760, and 0.521, respectively). Plasma IgM was significantly different among treatments, with its higher concentrations measured in fish fed 50% DMM and 100% DMM compared to those fed the control diet (100% FM; *P* < 0.05). In addition, neither MDA content nor the activities of SOD and GPx were significantly different between the dietary treatments (ANOVA, *P*=0.986, 0.383, and 0.322, respectively).

### 3.4. Digesta Microbiome

All 12 feed samples had less than 1,000 sequences after filtering and quality control, thus they were not analyzed further. The loss of sequences was mainly due to the filtering of chloroplast sequences resulting in an average loss of 19,615 sequences or a reduction of 97% of sequences on average (*Supplementary 1*). Digesta samples lost on average 408 sequences or a 6.5% reduction of sequences. After digesta samples (*n* = 32) were filtered of ASVs with less than 10 occurrences, there were 302,661 sequences distributed across 846 ASVs (see *Supplementary 1*) for a taxonomic rank breakdown).

**Figure 4:**
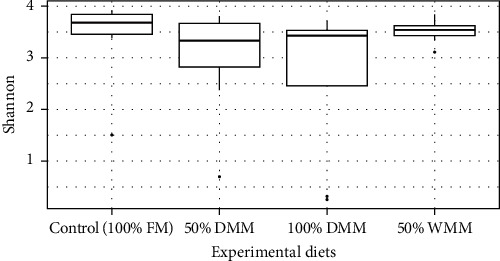
Boxplots showing digesta Shannon diversity by experimental diet. Bars denote largest and smallest values within 1.5 times the interquartile range while dots represent values outside that range. The middle line is the medium and ends of boxes are the first and third quartiles.

**Figure 5:**
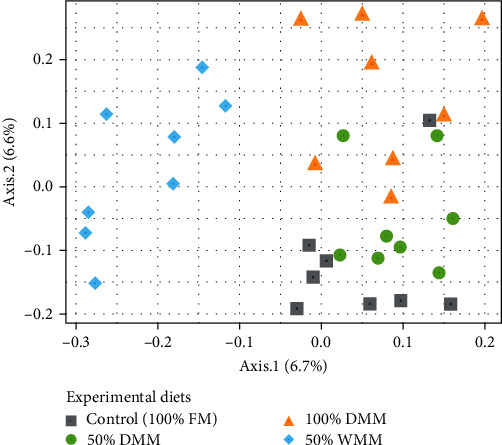
Principal coordinates of analysis of Bray–Curtis distances between samples. Shape and color represent the experimental diet. FM stands for fishmeal, DMM stands for defatted mealworm meal, and WMM stands for whole mealworm meal.

The Shannon diversity in digesta samples was statistically similar between all treatments based upon Welch's analysis of variance (Welch's ANOVA, *F* = 1.2974, *P*=0.3182) and pairwise using the Games–Howell test (adjusted *P*-values > 0.05) (Figure 4). On average, the Shannon diversity was 3.15 ± 1.0 across all samples. In terms of beta diversity, PERMANOVA results indicated a statistically significant difference across all diets (PERMANOVA, Monte Carlo *P*=0.001). Pairwise PERMANOVAs revealed that there was significant difference between 50% WMM and 100% FM as well as 100% DMM (PERMANOVA, Monte Carlo *P*=0.025 and 0.049, respectively; *Supplementary 1*). This is reflected in the PCoA which shows all 50% WMM samples clustering away from the other three diets (Figure 5).

**Figure 6:**
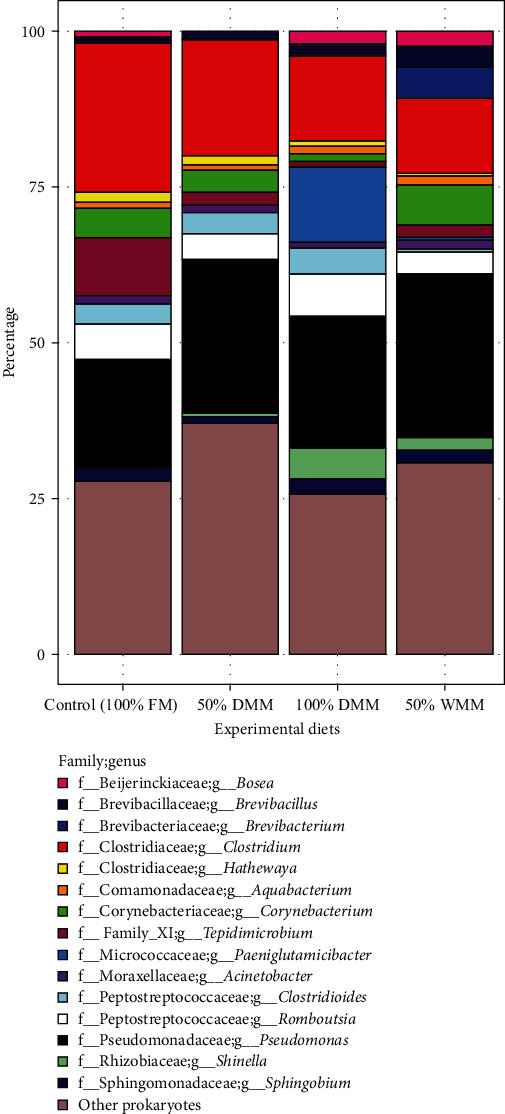
Stacked bar graphs showing the genera, which are represented by color, that had a mean relative abundance greater than 1% across all samples summarized by experimental treatment.

The most common phylum associated with the samples shifted from Firmicutes in 100% FM, 50% DMM, and 100% DMM to Proteobacteria in 50% WMM (*Supplementary 2*). This diet also had the highest percentage of Actinobacteriota. The most common genus in all diets besides 100% FM was *Pseudomonas* followed by *Clostridium*; this order was reversed in 100% FM (Figure 6). ANCOMBC2 analysis revealed that *Brevibacterium* (log fold change = 3.48 ± 1.05 standard error; ANCOMBC2, Holm *Q* = 0.021) and *Brachybacterium* (log fold change = 3.40 ± 1.04 standard error; ANCOMBC2, Holm *Q* = 0.024) were significantly more abundant in 50% WMM than 100% FM. *Tepidimicrobium* was significantly lower in 100% DMM than in 100% FM (log fold change = −3.60 ± 0.91 standard error; ANCOMBC2, Holm *Q* = 0.002).

Besides *Pseudomonas*, genera that have potentially pathogenic members for Atlantic salmon were not detected in this dataset including *Francisella* (associated disease = Francisellosis), *Aeromonas* (Furunculosis), *Renibacterium* (bacterial kidney disease), *Tenacibaculum* (tenacibaculosis), and *Vibrio* (vibriosis) [69–73]. None of the genera associated with epitheliocytis (*Piscichlamydia, Branchiomonas, Sygnamidia*, and *Clavochlamydia*) were detected in this study either [74]. *Flavobacterium* was detected in three samples, but the species was unknown [74]. Known lactic acid bacteria, a class of bacteria which have been shown to have positive benefits on gut health in general, that were not detected included: *Lactobacillus, Pediococcus, Leuconostoc, Aerococcus, Enterococcus, Vagococcus*, and *Carnobacterium* [75]. *Lactococcus* was detected in two subsamples (2-1FE and 2-2FE; both 50% WMM), and *Streptococcus* was detected in one subsample (12-1FE; 100% FM).

### 3.5. Relative Expression of Growth and Immunity-Related Genes in Intestine

**Figure 7:**
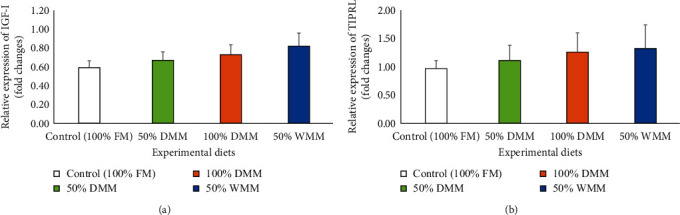
Effects of FM substitution by mealworm meals (at 50% DMM, 100% DMM, and 50% WMM) on the relative gene expression of insulin-like growth factor I (IGF-I) (a) and target of rapamycin signaling pathway regulator-like (TIPRL) (b) in Atlantic salmon fed the experimental diets for 12 weeks. Values are means with standard errors represented by vertical bars (*n* = 12). FM, fishmeal; DMM, defatted mealworm meal; and WMM, whole mealworm meal.

**Figure 8:**
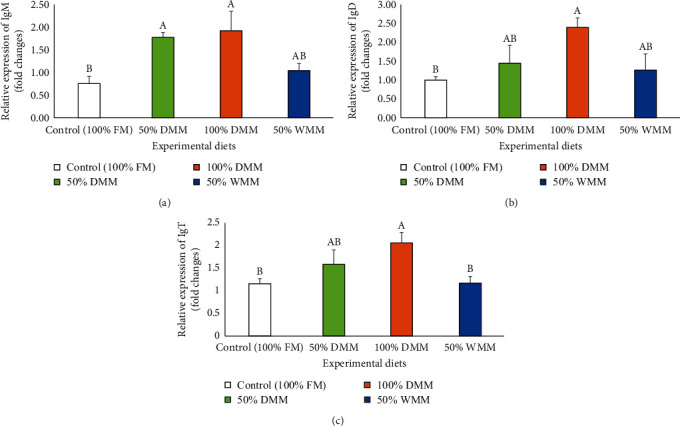
Effects of FM substitution by mealworm meals (at 50% DMM, 100% DMM, and 50% WMM) on the relative gene expression of immunoglobulin M (IgM) (a), immunoglobulin D (IgD) (b), and immunoglobulin T (IgT) (c) in Atlantic salmon fed the experimental diets for 12 weeks. Values are means with standard errors represented by vertical bars (*n* = 8). FM, fishmeal; DMM, defatted mealworm meal; and WMM, whole mealworm meal. Mean values with different letters are significantly different (*P* < 0.05).

Relative expressions of growth and immune-related genes which included insulin-like growth factor-I (IGF-I), target of rapamycin signaling pathway regulator-like (TIPRL), immunoglobulin M (IgM), immunoglobulin D (IgD), and immunoglobulin T (IgT) were analyzed in the intestine of Atlantic salmon and are presented in Figures 7 and 8. The relative expressions of IGF-I and TIPRL genes showed upregulation trends with the dietary substitutions and with higher levels in fish fed 100% DMM and 50% WMM but these increases were not statistically significant (ANOVA, *P*=0.473 and 0.853, respectively; Figures 7(a) and 7(b)). The relative gene expression of IgM was significantly different among treatments (*P* < 0.01) with higher levels observed in the groups fed 50% and 100% DMM diets compared to those fed the control (100% FM) diet (*P* < 0.05, Figure 8(a)). IgD gene expression of fish fed 100% DMM was significantly higher than those fed the control diet (*P* < 0.05, Figure 8(b)). The relative gene expression of IgT significantly increased in treatment with 100% DMM in contrast to the control and 50% WMM (*P* < 0.05, Figure 8(c)).

## 4. Discussion

Mealworms are rich in protein and lipids and appear to have amino acid and fatty acid compositions that should be nutritional adequate for many cultured finfish species [8]. As a result, several studies have been reported in the diets of finfish including rainbow trout [9, 10], gilthead seabream [15], European sea bass [13, 14], African catfish [16], common catfish, *Ameiurus melas* [18], yellow catfish [19], and Tilapia [20]. This study reports the substitution of fishmeal by mealworm meals both defatted and whole products with high-protein quality (both growth and health benefits) in the diet of Atlantic salmon using growth performance, feed utilization, feces stability, body composition, health parameters, digesta microbiome, and the relative expression of target genes.

The results of this study showed that fish fed diets produced with mealworm meals had similar growth metrics (final weight and weight gain), feed utilization, and protein efficiency when compared to those fed a control diet produced with a high-quality fishmeal. These results are in accordance with the previous reports of complete substitution of fishmeal by insect meals even in the diets of carnivorous finfish [5, 76–79]. In addition, the present study showed that whole-body proximate composition (crude protein, crude fat, fiber), whole-body amino acids, essential amino acids (threonine, valine, methionine, isoleucine, leucine, phenylalanine, lysine, histidine, arginine, and tryptophan) and taurine (conditional essential amino acid) were similar among treatments. Taken as a whole, these results suggest that the mealworm meals tested in this study provided adequate levels of nutrients needed to support protein metabolism (i.e., synthesis of proteins and amino acids) and growth of Atlantic salmon.

Feces stability is believed to be affected by the feed ingredients used because they may contain natural surfactants or other compounds that relate to particle binding or breakdown [42]. In this study, the particle size distributions of feces breakdown products were not affected by dietary treatments. However, it should be noted that the diets used in this study contained 0.3% gum guar as a binding agent [43] and the results should be viewed in this context. These results suggest that neither of the mealworm meals used in this study facilitated breakdown of the feces when mechanically agitated. From this perspective, mealworm meals appear to be suitable for use in RASs when included in stabilized feeds.

In this study, the whole-body essential fatty acids (EPAs) were influenced by the substitution of fishmeal with 100% mealworm meal. The results showed that EPA, linolenic, clupanodonic, homo-*α*-linolenic were significantly influenced by the dietary substitutions, with lower values in fish fed 100% DMM. However, DHA content in the whole body of Atlantic salmon was not significantly different between the treatments. In general, the differences observed can be attributed to the fact that the diets were formulated to be isolipidic but the fatty acid content was allowed to vary so long as similar levels of EPAs (DHA, EPA, and ARA) were obtained in the experimental diets. To obtain these concentrations, the high levels of lipid provided by the whole mealworm meal (50% WMM) were offset by reducing the amount of poultry oil added to this diet. Similarly, less fish oil was added to the fishmeal-based diet (100% FM) since fishmeal contains notable levels of EPAs (including DHA and EPA). Likewise, the observed differences should be viewed as oil blends that impacted the whole-body fatty acid concentrations measured in the salmon. As reviewed by Tran et al. [4], the concentrations of unsaturated fatty acids are high in mealworm and linoleic acid (18:2*n*-6) concentrations are much greater than that of *α*-linolenic acid (18:3*n*-3). In contrast to fish oil, terrestrial insects contain greater quantities of *n*-6 polyunsaturated fatty acids and negligible amounts of EPA (20:5*n*-3) and DHA (22:6*n*-3) [4]. It is also reported that salmonids can synthetize limited quantities of EPA and DHA from APA so dietary inclusion is more efficient [3]. Furthermore, one method to evaluate the adequacy of EFA provided in a diet is to evaluate the ratio of specific EFA in fish tissues with respect to the EFA provided by the diet, i.e., the fish to diet (FD) ratio [80]. This FD ratio could help to understand which EFA might be conserved, deficient, or selectively retained in terms of EFA in fish juveniles [32, 80]. In the present study, the FD ratios for the total omega-3 fatty acids and PUFA for all treatments were around 1 (≤1), suggesting that the requirements for EFAs were met when Atlantic salmon parr fed the mealworm diets as fishmeal does.

In fish nutrition studies, blood parameters including total protein, ALP, ALT, IgM, and TIBC are important indicators for health status in response to dietary manipulations of European sea bass [14], Indian major carps, *Labeo rohita*, *Catla catla*, and *Cirrhinus mrigala* [26], red seabream [27], blunt snout bream, *Megalobrama amblycephala* [35, 45, 81], largemouth bass, *Micropterus salmoides* [29, 31, 82], and Florida pompano, *Trachinotus carolinus* [32]. The total protein in plasma is related to the enhancement of digested protein [81, 83, 84]. The presence of ALP activity in plasma is directly related to the release of ALP enzymes from cells to the extracellular fluids [85, 86] and elevated activity of ALP may occur when there is cell growth, tissue necrosis, or leakage of ALP [86]. High plasma activity of ALT indicates a damage or weakening of normal liver function [32, 81, 87]. In the present study, plasma total protein, ALP, ALT, and TIBC levels were statistically similar among the dietary treatments. Immunoglobulin production is a specific immune response after being stimulated by antigen and the IgM class is the predominant immunoglobulin in fish [26]. In addition, IgM is not only the major antibody of primary response but also a key part of the adaptive immune response of fish [88]. In this study, plasma IgM levels were significantly higher in fish fed 50% DMM and 100% DMM when compared to those fed the control (100% FM diet). These results suggest that the substitution of fishmeal with mealworm meal in the diet of Atlantic salmon may result in health benefits through enhancing the adaptive immune system of Atlantic salmon. On the other hand, the elevated levels of IgM may indicate a specific immune response to the dietary ingredient. In either case, the results are in agreement with a previous study that reported plasma IgM levels increased significantly with increasing dietary mealworm contents in yellow catfish, *Pelteobagrus fulvidraco* [19]. More research is needed to evaluate the specific role and mechanisms of this response.

Fish liver typically has high concentrations of unsaturated fatty acids with a risk of oxidative damages that can result in the imbalance of reactive oxygen species (ROS) [89]. MDA is well-known as an oxidative stress marker [36, 90] that can be used to compare nutritional stress in fish. To overcome an oxidative stressor, fish are equipped with an antioxidant defense system to maintain endogenous ROS at a low level and attenuate oxidative damage resulting from high ROS reactivity [25, 36]. In this study, substitution of fishmeal with dietary mealworm meals did not influence the MDA content of Atlantic salmon suggesting that there was relatively low lipid peroxidation and, presumably, no oxidative damage. SOD, GPx, catalase, and glutathione are the key proteins in the antioxidants defense system [91] and their enzymatic activities can be correlated with fish nutritional factors [28, 31, 32, 35, 45, 82, 92]. In fish, SOD can catalyze dismutation of superoxide radicals to hydrogen peroxide that can be removed by GPx [36, 93, 94]. In the present study, SOD and GPx activities were not statistically affected by the substitution of mealworms meals indicating that antioxidative defense mechanisms were not activated which could be due to low levels of lipid peroxides as indicated by the measured MDA concentrations.

This study acknowledges that major differences exist between the transient and residential gut microbiomes, but some members of the community overlap, making comparisons possible [95]. Additionally, they are necessary in this case due to the limited examples of studies replacing fishmeal with mealworm conducting microbiome analysis. Other studies have also shown a dominance of Proteobacteria and Firmicutes at the phyla level as well as *Pseudomonas* and *Clostridium* at the general level as part of the normal microbiome in Atlantic salmon and other freshwater fish [96–99]. *Pseudomonas* members may be involved in protein utilization and cellular homeostasis allowing it to play a prominent role in ingestion performance [100]. On the other hand, some members of Pseudomonas such as *Pseudomonas anguilliseptica* are potential pathogens in Atlantic salmon [101]. Some *Clostridium* species such as *Clostridium butyricum* are considered mutualistic symbionts and/or probiotics due to their ability to supply fatty acids and vitamins to the host or stimulate the immune response, disease resistance, and enhance growth, respectively [97, 102–104].

There are conflicting reports in other studies into the replacement of fishmeal with *Tenebrio molitor* meal as to whether alpha diversity is statistically affected, which can be explained by differing fish physiology [105]. Shannon diversity was not statistically different in gilthead sea bream (*Sparus aurata*) or European sea bass (*Dicentrarchus labrax*) but was lower in rainbow trout (*Oncorhynchus mykiss*) when 50% of the fishmeal was replaced with *Tenebrio molitor* [40]. Furthermore, 25%, 50%, and 75% replacement of fishmeal with *Tenebrio molitor* did not change the Shannon diversity in European perch (*Perca fluviatilis*) [106]. In contrast to the above study, 100% replacement of fishmeal with *Tenebrio molitor* in the diet of rainbow trout (*O. mykiss*) led to a higher Shannon diversity in the intestinal mucosa microbiome [41]. Changes in richness can be associated with negative consequences such as dysbiosis or loss of functional redundancy. It could be considered a positive result that this study shows no significant differences between the diets in terms of Shannon diversity.

When considering the beta diversity, a significant shift in the microbiome was found between fish fed 50% WMM and the control (100% FM) diets and between 50% WMM and 50% DMM. Significant differences in beta diversity or in specific genera between insect meal diets and the control have been noted in other studies [40, 41, 106]. The addition of defatted mealworm (*Tenebrio molitor*) to the diet of European perch (*Perca fluviatilis*) led to significantly reduced relative percentages of *Lactobacillus* and *Streptococcus* [106]. Although no differentially abundant genera were noted in rainbow trout fed insect meal diets [41]. Both *Lactobacillus* and *Streptococcus* were not detected in most samples in this study, although other genera were shown to be differentially abundant between the control (100% FM) and mealworm meal diets. These included *Brevibacterium, Brachybacterium*, and *Tepidimicrobium*. *Brevibacterium* has been found to be enriched in a black soldier fly prepupae meal diet in comparison to a control diet in rainbow trout [107]. *Brevibacterium* members are Gram-positive, obligate aerobes that can survive carbohydrate starvation and reduce nitrates to nitrites [108]. It has been identified as a potential probiotic due to its potential contribution to nutritional processes in Artic charr (*Salvelinus alpinus*) [109]. This is a positive change in the microbiome that is statistically higher in the 50% WMM diet in comparison to the 100% FM diet in this study. Brachybacterium members are Gram-positive, aerobes that can grow slowly in anaerobic environments and have been isolated from the gut of several fish including Atlantic salmon [110, 111]. Although the positive effects of any members of these genera have not been explored as a probiotic, a study showed that an exopolysaccharide isolated from a strain of Brachybacterium isolated from Asian seabass exhibited antibacterial activity and suggested further exploration as a source of marine drugs [112]. No conclusions could be made regarding whether its increased presence in fish fed 50% WMM could be considered a positive or negative. *Tepidimicrobium* species are mostly strict or facultative anaerobes with broad fermentative capabilities that are capable of Fe(III) reduction [113]. Since it has been identified as a potential commensal species in Atlantic salmon due to its broad fermentative capabilities, its decrease in the 100% DMM samples may be considered a negative if other potential commensals do not overcome its absence [114].

The organ growth of fish is controlled by the endocrine system, particularly through the growth hormone—insulin-like growth factor (IGF) axis [115]. Target of rapamycin pathway (TOR pathway) regulates protein synthesis genes [31, 35, 36]. In this study, no statistical differences were observed in the relative expressions of IGF-I and TIPRL genes among treatments, consistent with the similarity in growth metrics among treatments. Immune response (activation of the immune system) comes either from the imbalance between supply and utilization of nutrients or nutritional and environmental stressors [36]. In teleosts, three classes of immunoglobulins (Igs) have been identified, such as IgM, IgD, and IgT, with IgM+ being the predominant surface Ig isotype [33, 34]. IgM and IgD isotypes are evolutionary conserved and present in all teleost species, IgT is only found in some teleosts, including Atlantic salmon [116, 117]. In this study, the relative expression of IgM gene was significantly upregulated in the groups fed 50% and 100% DMM diets compared to those fed the control diet. IgD gene expression of fish fed 100% DMM was significantly higher than the control. The relative gene expression of IgT significantly increased in treatment with 100% DMM in contrast to the control and 50% WMM. These immunoglobulin genes support the plasma IgM results in this study. Su et al. [19] also reported significant upregulation of IgM-related genes in yellow catfish fed with mealworm diets. The immunomodulatory functions could be due to chitin availability in insects including mealworms [14, 21, 23]. Those signaling molecules help in understanding the specific mechanisms behind fish growth and health benefits of mealworm meal that guide future research directions of Atlantic salmon (finfish), as well as its responses to dietary substitutions.

## 5. Conclusions

Across all treatments, Atlantic salmon in this study showed high survival, and no significant differences were observed in growth performance, feces stability, body composition (except unsaturated fatty acids), health parameters (except IgM), the relative expression of growth-related genes, and alpha diversity of digesta microbiome. The most common genus in all treatments was *Pseudomonas*, which has been previously reported to have both commensal and pathogenic members. Plasma IgM content and intestinal immune genes (IgM, IgD, and IgT) expression were significantly upregulated by the dietary substitutions of defatted mealworm meal, suggesting that mealworm substitution in the diet of Atlantic salmon could increase health benefits through enhancing the adaptive immune system. Overall, the mealworm meals performed well indicating similar growth, survival, and health benefits to fishmeal. The findings of this study provide valuable information and insights for studies on fish nutrition, particularly those geared toward the optimization of nutritionally balanced (healthy), cost-effective, and environment-friendly commercial feeds for Atlantic salmon and other farmed species.

## Figures and Tables

**Table 1:** Nutrient composition of test ingredients (mealworm meals) ^*∗*^.

Nutritional value	WMM	DMM
Energy (kcal/kJ)	510/2142	554/2317
Crude protein (%)	59.6	70.0
Crude fat (%)	28.7	10.0
Fibers (%)	3.7	8.0
Ashes (%)	—	3.5
Moisture	5.0	4.0
Total amino acids (g/100 g)		
Alanine	4.0	4.9
Arginine	3.2	3.5
Aspartic acid	5.3	5.8
Cysteine + cystine	0.6	0.4
Glutamic acid	7.2	8.0
Glycine	2.8	3.6
Histidine	2.0	1.9
Isoleucine	2.6	2.9
Leucine	4.0	5.0
Lysine	3.9	3.7
Methionine	0.8	0.8
Phenylalanine	2.6	2.6
Proline	3.7	4.6
Serine	2.5	3.1
Threonine	2.4	2.8
Tryptophan	0.7	0.8
Tyrosine	4.4	4.9
Valine	3.4	4.2
Fatty acids (percentage of total fats)		
C12:0 (lauric acid)	—	0.4
C14:0 (myristic acid)	—	3.0
C16:0 (palmitic acid)	25.8	17.0
C18:0 (stearic acid)	7.1	4.2
C18:1-*n*9c (oleic acid)	31.7	40.3
C18:2-*n*6c (linoleic acid)	28.9	29.9
C18:3-*n*3 (alpha-linolenic acid)	1.8	1.0
Saturated fatty acids	34.7	25.4
Sum monosaturated fatty acids (MUFA)	34.3	42.6
Sum polyunsaturated fatty acids (PUFA)	31.0	31.3
Total trans fatty acids	1.2	0.6
Fatty acids sum of omega 3 calc.	1.8	1.1
Fatty acids sum of omega 6 calc.	29	29.9
Minerals (mg/kg)		
Calcium (Ca)	520	633.5
Phosphorus (P)	7,633.0	9,125.0
Potassium (K)	9,600.0	10,335.0
Magnesium (Mg)	1,150	2,912.5
Iron (Fe)	51.0	75.6
Copper (Cu)	19.0	24.0
Manganese (Mn)	5.9	15.2
Zinc (Zn)	135.0	—

^*∗*^Source: Ÿnsect France. WMM, whole mealworm meal (*Alphitobius diaperinus*); DMM, defatted mealworm meal (*Tenebrio molitor*).

**Table 2:** Formulation and composition of the experimental diets^1^.

Ingredients	Control diet (100% FM)	Test diets (g/100 g diet) ^*∗*^		
		50% DMM	100% DMM	50% WMM
Fishmeal (SeaPro^TM^ 75) ^*∗∗*^	20.0	10.0	0.0	10.0
Defatted mealworm meal	0.0	10.7	21.5	0.0
Whole mealworm meal	0.0	0.0	0.0	12.6
Poultry meal, pet food grade	15.0	15.0	15.0	15.0
Soybean meal	3.0	3.0	3.0	3.0
Soy protein concentrate 1	8.9	8.9	8.9	8.9
Corn protein concentrate (75% CP)	5.2	5.2	5.2	5.2
Wheat gluten meal	3.5	3.5	3.5	3.5
Wheat flour 70% starch 2	18.5	18.1	17.2	18.5
Fish oil	12.7	13.4	14.2	14.3
Poultry oil	6.0	6.0	6.0	1.8
Vitamin premix ARS 702	1.5	1.5	1.5	1.5
Stay C 35%	0.2	0.2	0.2	0.2
Choline chloride	0.6	0.6	0.6	0.6
Trace mineral premix ARS 1440	0.1	0.1	0.1	0.1
Lysine HCl	1.3	1.3	1.3	1.3
DL-methionine	0.5	0.5	0.5	0.5
Taurine	0.5	0.5	0.5	0.5
Astaxanthin	0.1	0.1	0.1	0.1
Gum guar	0.3	0.3	0.3	0.3
*α*-Cellulose	2.1	1.0	0.5	2.1
Total	100.0	100.0	100.0	100.0
Diet composition (calculated, in dry matter)				
Crude protein (CP, %)	43.8	43.7	43.6	43.8
Lipid (%)	22.5	22.5	22.5	22.7
Digestible energy (kcal/100 g)	431.9	430.5	427.4	433.6
Fiber (%)	2.4	2.1	2.3	2.7
Lysine (Lys, %)	3.2	3.1	3.0	3.2
Methionine (Met, %)	1.3	1.2	1.1	1.2
Total sulfur amino acids (TSAA, %)	1.9	1.8	1.7	1.8
Threonine (Thr, %)	1.8	1.7	1.6	1.7
Arginine (Arg, %)	2.7	2.5	2.3	2.5
Taurine (Tau, %)	0.5	0.5	0.5	0.5
Phosphorus (P)	2.2	2.2	2.3	2.2

^1^Extruded diets were produced at the USDA—ARS facility in Bozeman, MT, USA.  ^*∗*^All substitutions were done on a crude protein basis. FM, fishmeal; DMM, defatted mealworm meal (*Tenebrio molitor*); WMM, whole mealworm meal (*Alphitobius diaperinus*).  ^*∗∗*^SeaPro^TM^ 75 is a deboned, high-protein (75% CP), low-ash fishmeal derived from fresh cuttings of marine whitefish; it provides a supplemental level of omega-3 fatty acids including DHA and EPA; and it is stabilized with natural antioxidants (BioOregon Protein Inc., Warrenton, OR, USA).

**Table 3:** Analyzed proximate and amino acids composition of the experimental diets.

Proximate composition (%, as is)	Diets (%)			
	Control (100% FM)	50% DMM	100% DMM	50% WMM
Crude protein ^*∗*^	44.80	45.90	45.30	46.30
Moisture	4.00	3.50	3.50	3.00
Crude fat	20.89	21.33	23.28	20.38
Crude fiber	2.27	2.62	3.28	3.33
Ash	5.28	5.02	5.58	4.86
Essential amino acids (W/W%)				
Threonine	1.64	1.60	1.57	1.63
Valine	2.17	2.26	2.39	2.28
Methionine	1.43	1.36	1.21	1.35
Isoleucine	2.03	2.03	2.02	2.08
Leucine	3.64	3.64	3.61	3.68
Phenylalanine	2.02	2.02	2.01	2.12
Lysine	3.78	3.68	3.43	3.69
Histidine	0.99	1.05	1.12	1.08
Arginine	2.52	2.47	2.41	2.52
Tryptophan	0.46	0.44	0.46	0.43
Conditional essential amino acid (W/W%)				
Taurine^§^	0.69	0.70	0.67	0.69
Nonessential amino acids (W/W%)				
Hydroxyproline	0.34	0.36	0.33	0.34
Aspartic acid	3.75	3.64	3.50	3.67
Serine	1.66	1.64	1.68	1.67
Glutamic acid	7.70	7.53	7.32	7.91
Proline	2.55	2.80	3.05	2.89
Lanthionine^§^	0.17	0.17	0.18	0.19
Glycine	2.30	2.38	2.45	2.37
Alanine	2.41	2.55	2.73	2.58
Cysteine	0.59	0.58	0.57	0.60
Tyrosine	1.48	1.73	2.03	1.73
Hydroxylysine	0.04	0.03	0.03	0.04
Ornithine^§^	0.11	0.07	0.08	0.09
Total AAs (W/W%)	44.41	44.68	44.82	45.58

Analyses (except crude protein and ash) were carried out by the Experiment Station Chemical Laboratories (ESCL), University of Missouri, Columbia, MO, USA. Crude protein analysis was conducted at USDA—ARS facility, Franklin, ME, USA. W/W% = g per 100 g of sample. Crude protein ^*∗*^ = %*N* × 6.25. ^§^Nonproteinogenic amino acids. Results are expressed on an “as is” basis.

**Table 4:** Analyzed fatty acids composition of the experimental diets.

Fatty acids profile	Diets (%)			
	Control (100% FM)	50% DMM	100% DMM	50% WMM
C14:0	1.54	1.54	1.60	1.66
Myristoleic (9c-14:1)	0.08	0.07	0.07	0.06
C15:0	0.18	0.18	0.18	0.20
C15:1*n*5	0.01	0.00	0.00	0.01
Palmitic (16:0)	19.97	19.54	19.85	20.71
Palmitoleic (9c-16:1)	5.06	4.59	4.79	4.48
Margaric (17:0)	0.20	0.22	0.21	0.23
10c-17:1	0.24	0.23	0.21	0.24
Stearic (18:0)	4.70	4.76	4.87	5.10
Elaidic (9t-18:1)	0.23	0.21	0.27	0.19
Oleic (9c-18:1)	25.26	25.34	26.80	24.76
Vaccenic (11c-18:1)	3.87	4.15	3.57	4.44
Linoelaidic (18:2t)	0.04	0.03	0.04	0.02
Linoleic (18:2*n*6)	12.88	14.64	14.40	10.55
Linolenic (18:3*n*3)	**1.22**	**1.25**	**1.10**	**1.08**
g-Linolenic (C18:3*n*6)	0.11	0.11	0.10	0.07
Stearidonic (18:4*n*3)	**0.00**	**0.63**	**0.00**	**0.74**
Arachidic (20:0)	0.11	0.14	0.14	0.17
Gonodic (20:1*n*9)	0.76	0.76	0.67	0.83
C20:2	0.24	0.28	0.06	0.29
Homo-g-linolenic (C20:3*n*6)	0.12	0.11	0.10	0.08
Homo-a-linolenic (20:3*n*3)	**0.11**	**0.09**	**0.10**	**0.11**
ARA (20:4*n*6)	0.81	0.75	0.65	0.62
EPA (20:5*n*3)	**8.24**	**7.74**	**7.26**	**9.03**
C21:0	0.03	0.03	0.01	0.03
Behenoic (22:0)	0.07	0.02	0.08	0.03
Erucic (22:1*n*9)	0.15	0.15	0.14	0.17
C22:2*n*6	0.05	0.05	0.05	0.05
Adrenic (C22:4*n*6)	0.10	0.10	0.09	0.06
C22:5*n*6 ^*∗*^	0.12	0.10	0.09	0.10
Clupanodonic (22:5*n*3)	**1.27**	**1.23**	**1.18**	**1.41**
DHA (22:6*n*3)	**5.36**	**4.90**	**4.33**	**5.54**
C23:0	0.04	0.04	0.03	0.05
Lignoceric (24:0)	0.03	0.04	0.04	0.04
Nervonic (24:1*n*9)	0.24	0.18	0.20	0.19

Analyses were carried out by the Experiment Station Chemical Laboratories (ESCL), University of Missouri, Columbia, MO, USA. W/W% = g per 100 g of sample.  ^*∗*^Omega-3 fatty acids are in bold type. Results are expressed on an “as is” basis unless.

**Table 5:** Primers sequences for real-time qPCR.

Target gene	Primer sequences (5′–3′)		Accession number	Product size (bp)	Source
	Forward	Reverse			
IGF-I	TGGGGATGTCTAGCGGTCAT	AGTGAGAGGGTGTGGGTACA	XM_014208346.2	94	New design
TIPRL	CTGCACGACCACGGAGTATC	CTCCATCCACTCGCAGGAAG	XM_014136761.2	97	New design
IgM	AGGCGGAAATTCCCTGACTG	CACGGAGTTGACTGACTCCC	Y12457.1	83	New design
IgD	CGTCTACTCCATCGCTCCAC	TTTGGCGTCATACGCAGAGT	AF141607.1	104	New design
IgT	CAAAGGGCAACCTGAACAGC	GAACGACCGGTGTGTCTTCA	GQ907004.1	117	New design
*β*-actin	CCAAAGCCAACAGGGAGAA	AGGGACAACACTGCCTGGAT	BG933897	102	[59]

IGF-I, insulin-like growth factor-I; TIPRL, target of rapamycin signaling pathway regulator-like; IgM, immunoglobulin M; IgD, immunoglobulin D; IgT, immunoglobulin T; *β*-actin, beta-actin (reference gene). The primers of target genes were designed using online resources according to the partial cDNA sequences of the target genes using Atlantic salmon (*Salmo salar*) transcriptome analysis. All primers of the target genes and reference genes were synthesized by Integrated DNA Technologies (IDT, Morrisville, NC, USA).

**Table 6:** Growth performance, survival, feed utilization, and condition indices of Atlantic salmon fed the experimental diets for 12 weeks.

Parameters	Diets			
	Control (100% FM)	50% DMM	100% DMM	50% WMM
Initial weight (g)	38.5 ± 0.1	38.7 ± 0.1	38.4 ± 0.1	38.4 ± 0.2
Survival (%)	100.0 ± 0.0	100.0 ± 0.0	98.8 ± 2.5	100.0 ± 0.0
Final weight (g)	182.8 ± 5.5	179.0 ± 2.6	191.4 ± 5.5	191.5 ± 3.6
Weight gain (%)	374.7 ± 14.4	363.4 ± 6.2	392.7 ± 9.6	398.8 ± 8.3
Feeding rate (%)	1.53 ± 0.05	1.50 ± 0.04	1.50 ± 0.00	1.60 ± 0.00
Feed efficiency ratio	1.23 ± 0.03	1.20 ± 0.00	1.25 ± 0.03	1.23 ± 0.03
PER (%)	2.64 ± 0.06	2.54 ± 0.03	2.69 ± 0.03	2.57 ± 0.04
K-factor (%)	1.14 ± 0.02	1.19 ± 0.02	1.18 ± 0.02	1.25 ± 0.08
VSI (%)	10.00 ± 0.20	9.83 ± 0.28	9.61 ± 0.23	9.89 ± 0.19
HSI (%)	1.48 ± 0.05^a^	1.43 ± 0.05^ab^	1.28 ± 0.04^bc^	1.24 ± 0.05^c^

PER, protein efficiency ratio; K-factor, Fulton condition factor; VSI, viscerosomatic index; HSI, hepatosomatic index. Mean values within a row with different superscript letters were significantly different (*P* < 0.05).

**Table 7:** Whole-body proximate and amino acids composition of Atlantic salmon fed the experimental diets for 12 weeks.

Proximate composition (as is)	Initial	Diets			
		Control (100% FM)	50% DMM	100% DMM	50% WMM
Crude protein ^*∗*^	54.84	51.34 ± 0.76	52.40 ± 0.37	51.35 ± 0.44	51.91 ± 1.44
Moisture	5.78	4.27 ± 0.56	4.48 ± 0.56	3.89 ± 0.31	3.98 ± 0.54
Crude fat	31.44	39.27 ± 0.66	37.66 ± 0.67	39.12 ± 0.87	37.22 ± 0.78
Fiber	0.15	0.06 ± 0.04	0.10 ± 0.04	0.14 ± 0.07	0.11 ± 0.02
Ash	7.23	4.84 ± 0.18	5.11 ± 0.26	5.26 ± 0.09	5.60 ± 0.44
Essential amino acids (W/W%)					
Threonine	2.27	2.27 ± 0.04	2.31 ± 0.03	2.28 ± 0.03	2.32 ± 0.03
Valine	2.66	2.70 ± 0.05	2.76 ± 0.05	2.69 ± 0.03	2.75 ± 0.03
Methionine	1.56	1.53 ± 0.02	1.55 ± 0.03	1.56 ± 0.02	1.56 ± 0.02
Isoleucine	2.33	2.35 ± 0.07	2.42 ± 0.04	2.33 ± 0.05	2.41 ± 0.03
Leucine	3.75	3.77 ± 0.07	3.84 ± 0.06	3.72 ± 0.07	3.83 ± 0.05
Phenylalanine	2.09	2.04 ± 0.04	2.09 ± 0.03	2.06 ± 0.03	2.09 ± 0.03
Lysine	4.34	4.47 ± 0.10	4.56 ± 0.09	4.40 ± 0.06	4.55 ± 0.05
Histidine	1.32	1.30 ± 0.02	1.33 ± 0.02	1.31 ± 0.01	1.33 ± 0.01
Arginine	3.12	2.96 ± 0.08	3.07 ± 0.03	3.23 ± 0.20	3.09 ± 0.05
Tryptophan	0.52	0.56 ± 0.02	0.59 ± 0.02	0.56 ± 0.02	0.59 ± 0.02
Conditional essential amino acid (W/W%)					
Taurine^§^	0.48	0.12 ± 0.11	0.01 ± 0.00	0.01 ± 0.00	0.14 ± 0.13
Nonessential amino acids (W/W%)					
Hydroxyproline	0.49	0.31 ± 0.01	0.34 ± 0.03	0.56 ± 0.21	0.41 ± 0.05
Aspartic acid	4.85	4.74 ± 0.09	4.83 ± 0.07	4.82 ± 0.09	4.86 ± 0.07
Serine	1.86	1.81 ± 0.02	1.82 ± 0.01	1.86 ± 0.08	1.83 ± 0.04
Glutamic acid	6.46	6.31 ± 0.09	6.27 ± 0.10	6.27 ± 0.15	6.34 ± 0.12
Proline	2.04	1.78 ± 0.06	1.83 ± 0.02	2.13 ± 0.31	1.87 ± 0.04
Lanthionine^§^	0.25	0.27 ± 0.09	0.36 ± 0.02	0.36 ± 0.02	0.34 ± 0.01
Glycine	3.53	2.79 ± 0.07	2.87 ± 0.03	3.57 ± 0.69	2.99 ± 0.09
Alanine	3.10	2.95 ± 0.05	3.01 ± 0.03	3.16 ± 0.20	3.04 ± 0.05
Cysteine	0.55	0.58 ± 0.01	0.58 ± 0.01	0.59 ± 0.01	0.59 ± 0.01
Tyrosine	1.94	2.10 ± 0.03	2.18 ± 0.02	2.12 ± 0.05	2.18 ± 0.02
Hydroxylysine	0.07	0.04 ± 0.01	0.04 ± 0.00	0.07 ± 0.00	0.04 ± 0.00
Ornithine^§^	0.06	0.05 ± 0.01	0.06 ± 0.00	0.05 ± 0.00	0.06 ± 0.00
Total AAs (W/W%)	49.64	47.76 ± 0.86	48.70 ± 0.57	49.67 ± 1.87	49.18 ± 0.80

Analyses were carried out by the Experiment Station Chemical Laboratories (ESCL), University of Missouri, Columbia, MO, USA. W/W% = g per 100 g of sample. Crude protein ^*∗*^ = %*N* × 6.25. ^§^Nonproteinogenic amino acids. Results are expressed on an “as is” basis. Mean values within a row with different superscript letters were significantly different (*P* < 0.05).

**Table 8:** Whole-body fatty acids composition of Atlantic salmon fed the experimental diets for 12 weeks.

Fatty acid composition	Initial	Diets			
		Control (100% FM)	50% DMM	100% DMM	50% WMM
C14:0	4.69	1.84 ± 0.03	1.93 ± 0.03	2.09 ± 0.08	2.04 ± 0.08
Myristoleic (9c-14:1)	0.07	0.06 ± 0.00^a^	0.06 ± 0.00^ab^	0.07 ± 0.00^a^	0.05 ± 0.00^b^
C15:0	0.37	0.18 ± 0.00	0.18 ± 0.00	0.20 ± 0.01	0.20 ± 0.01
Palmitic (16:0)	19.34	17.39 ± 0.21	17.37 ± 0.24	18.21 ± 0.59	18.13 ± 0.46
Palmitoleic (9c-16:1)	7.79	5.60 ± 0.06	5.54 ± 0.13	5.92 ± 0.23	5.52 ± 0.12
Margaric (17:0)	0.34	0.18 ± 0.00^b^	0.19 ± 0.00^ab^	0.20 ± 0.01^ab^	0.21 ± 0.01^a^
10c-17:1	0.27	0.25 ± 0.00^b^	0.25 ± 0.00^b^	0.26 ± 0.01^ab^	0.27 ± 0.00^a^
Stearic (18:0)	4.85	4.36 ± 0.07	4.31 ± 0.02	4.45 ± 0.07	4.52 ± 0.09
Elaidic (9t-18:1)	0.42	0.24 ± 0.01	0.20 ± 0.02	0.24 ± 0.01	0.20 ± 0.01
Oleic (9c-18:1)	24.51	27.68 ± 0.33^b^	26.86 ± 0.12^b^	29.29 ± 0.57^a^	27.87 ± 0.26^ab^
Vaccenic (11c-18:1)	4.11	4.31 ± 0.10	4.49 ± 0.14	4.51 ± 0.19	4.64 ± 0.12
Linoelaidic (18:2t)	0.03	0.02 ± 0.00	0.03 ± 0.00	0.03 ± 0.00	0.03 ± 0.00
Linoleic (18:2*n*6)	8.04	11.17 ± 0.09^b^	12.16 ± 0.11^a^	11.83 ± 0.17^a^	9.59 ± 0.03^c^
Linolenic (18:3*n*3)	**0.85**	**1.06 ± 0.01** ^a^	**1.09 ± 0.01** ^a^	**0.94 ± 0.03** ^b^	**0.97 ± 0.01** ^b^
g-Linolenic (C18:3*n*6)	0.17	0.17 ± 0.01	0.17 ± 0.01	0.17 ± 0.01	0.14 ± 0.00
Stearidonic (18:4*n*3)	**0.00**	**0.29 ± 0.17**	**0.60 ± 0.01**	**0.54 ± 0.03**	**0.49 ± 0.16**
Arachidic (20:0)	0.13	0.10 ± 0.01^b^	0.11 ± 0.01^ab^	0.12 ± 0.00^a^	0.12 ± 0.01^ab^
Gonodic (20:1*n*9)	1.31	1.25 ± 0.02	1.29 ± 0.06	1.26 ± 0.02	1.29 ± 0.03
C20:2	0.09	0.75 ± 0.01	0.84 ± 0.01	0.81 ± 0.01	0.53 ± 0.17
Homo-g-linolenic (C20:3*n*6)	0.25	0.29 ± 0.02	0.29 ± 0.03	0.24 ± 0.02	0.21 ± 0.01
Homo-a-linolenic (20:3*n*3)	**0.12**	**0.13 ± 0.00** ^a^	**0.13 ± 0.00** ^a^	**0.11 ± 0.01** ^b^	**0.13 ± 0.00** ^a^
Arachidonic (20:4*n*6)	0.62	0.70 ± 0.01^a^	0.70 ± 0.01^a^	0.54 ± 0.04^b^	0.59 ± 0.03^b^
EPA (20:5*n*3)	**3.73**	**4.62 ± 0.09** ^ab^	**4.64 ± 0.13** ^ab^	**3.76 ± 0.38** ^b^	**4.99 ± 0.27** ^a^
C21:0	0.03	0.02 ± 0.00	0.02 ± 0.00	0.023 ± 0.00	0.023 ± 0.00
Behenoic (22:0)	0.08	0.05 ± 0.00	0.04 ± 0.01	0.05 ± 0.01	0.06 ± 0.00
Erucic (22:1*n*9)	0.20	0.23 ± 0.04	0.23 ± 0.03	0.17 ± 0.00	0.20 ± 0.00
C22:2*n*6	0.12	0.12 ± 0.00	0.12 ± 0.00	0.10 ± 0.00	0.10 ± 0.00
Adrenic (C22:4*n*6)	0.09	0.12 ± 0.00^a^	0.11 ± 0.00^ab^	0.09 ± 0.01^bc^	0.09 ± 0.00^c^
C22:5*n*6	0.19	0.17 ± 0.00^a^	0.16 ± 0.01^a^	0.12 ± 0.01^b^	0.15 ± 0.01^ab^
Clupanodonic (22:5*n*3)	**1.33**	**2.10 ± 0.04** ^ab^	**2.03 ± 0.08** ^ab^	**1.63 ± 0.15** ^b^	**2.11 ± 0.15** ^a^
DHA (22:6*n*3)	**5.53**	**7.91 ± 0.14**	**7.75 ± 0.27**	**5.87 ± 0.72**	**7.56 ± 0.63**
C23:0	0.04	0.04 ± 0.01	0.05 ± 0.01	0.04 ± 0.01	0.06 ± 0.01
Lignoceric (24:0)	0.04	0.03 ± 0.01	0.02 ± 0.00	0.02 ± 0.00	0.02 ± 0.00
Nervonic (24:1*n*9)	0.44	0.28 ± 0.01	0.26 ± 0.02	0.24 ± 0.02	0.26 ± 0.03

Analyses were carried out by the Experiment Station Chemical Laboratories (ESCL), University of Missouri, Columbia, MO, USA. W/W% = g per 100 g of sample.  ^*∗*^Omega-3 fatty acids are in bold type. Results are expressed on an “as is” basis. Mean values within a row with different superscript letters were significantly different (*P* < 0.05).

**Table 9:** Plasma health parameters, and hepatic peroxide and antioxidants of Atlantic salmon fed the experimental diets for 12 weeks.

Parameters	Diets			
	Control (100% FM)	50% DMM	100% DMM	50% WMM
Plasma health parameters				
ALT (mU/ml)	8.93 ± 1.09	8.16 ± 1.11	8.75 ± 1.23	5.07 ± 0.85
ALP (U/l)	253.29 ± 35.97	246.32 ± 28.05	268.81 ± 24.48	299.91 ± 26.44
TP (g/l)	26.48 ± 3.45	30.42 ± 4.68	32.72 ± 4.09	31.03 ± 4.54
IgM (*µ*g/ml)	547.07 ± 17.66^b^	777.46 ± 82.72^a^	718.90 ± 57.60^a^	669.61 ± 40.87^ab^
TIBC (*µ*mol/l)	78.55 ± 7.48	86.55 ± 17.75	100.76 ± 7.63	80.58 ± 13.70
Hepatic peroxide and antioxidants				
MDA (nmol/mg)	27.56 ± 5.45	26.04 ± 3.62	27.75 ± 6.49	28.99 ± 6.32
SOD (U/mg)	1.70 ± 0.13	1.72 ± 0.20	1.33 ± 0.22	1.47 ± 0.17
GPx (U/ml)	0.89 ± 0.13	0.62 ± 0.12	0.90 ± 0.19	1.06 ± 0.18

ALT, alanine aminotransferase; ALP, alkaline phosphatase; TP, total protein; IgM, immunoglobulin M; TIBC, total iron-binding capacity; MDA, malondialdehyde; SOD superoxide dismutase; GPx, glutathione peroxidase. Mean values within a row with different superscript letters were significantly different (*P* < 0.05).

## Data Availability

The original contributions presented in the study are included in the article/supplementary material, further inquiries can be directed to the corresponding author.

## Supplementary Materials

Supplementary 1 Table S1: per sample sequence count changes over analysis stages. Table S2: total and named (does not have uncultured, unknown, etc… as part of name or is a repeat of the name of at the previous taxonomic level) taxa at each taxonomic level for digesta samples. Table S3: permutational analysis of variance results.

Supplementary 2 Figure S1: stacked bar graphs showing the phyla, which are represented by color, that had a mean relative abundance greater than 1% across all samples summarized by experimental treatment.
