# Supplementary material for: Effects of Fishmeal Substitution with Mealworm Meals (Tenebrio molitor and Alphitobius diaperinus) on the Growth, Physiobiochemical Response, Digesta Microbiome, and Immune Genes Expression of Atlantic Salmon (Salmo salar)
Source: Aquac Nutr. 2024 Jan 6;2024:6618117. doi: 10.1155/2024/6618117 (PMC10787657; doi:10.1155/2024/6618117)
Supplement: Supplementary 1 — Table S1: per sample sequence count changes over analysis stages. Table S2: total and named (does not have uncultured, unknown, etc… as part of name or is a repeat of the name of at the previous taxonomic level) taxa at each taxonomic level for digesta samples. Table S3: permutational analysis of variance results. [file 6618117.f1.pdf]

Supplementary Table 1. Per sample sequence count changes over analysis stages.

Sample ID	Raw	Post-Dada2	Post Filtering Mitochondria	Post Filtering Chloroplasts	Post Filtering Eukaryotes	Post Filtering Unknown	Post Filtering Low Abundance ASVs
10-1FE	52830	8699	8699	8615	8615	8615	8535
10-2FE	34518	4546	4524	4392	4392	4392	4235
11-1FE	52176	9366	9366	8090	8090	8090	8026
11-2FE	47536	5590	5574	5216	5216	5216	5168
1-1FE	63046	14042	14027	14019	14019	14019	13879
12-1FE	82012	19949	19949	19923	19923	19917	19822
12-2FE	49668	10918	10918	10409	10409	10409	10318
1-2FE	55578	11297	11297	10479	10479	10479	10398
13-1FE	35473	8188	8180	8049	8049	8049	7927
13-2FE	48620	10307	10307	9957	9957	9957	9892
14-1FE	102565	32723	32719	32415	32415	32386	32336
14-2FE	53194	7371	7216	6121	6121	6039	5957
15-1FE	27997	6487	6487	6158	6158	6158	6146
15-2FE	17167	2394	2394	2348	2348	2348	2307
16-1FE	83146	10434	10434	10348	10348	10348	10267
16-2FE	46136	3190	3181	2432	2432	2432	2380
2-1FE	63123	5824	5824	5418	5418	5418	5386
2-2FE	54091	3157	3152	2468	2468	2468	2409
3-1FE	31001	5278	5278	5214	5214	5214	5167
3-2FE	25667	2332	2332	2236	2236	2236	2186
4-1FE	35172	9836	9836	9341	9341	9341	9302
4-2FE	39699	10370	10370	10125	10125	10125	10060
5-1FE	41436	6124	6124	5612	5612	5612	5577
5-2FE	106260	35614	35614	35552	35552	35552	35503
6-1FE	40927	9796	9790	9452	9452	9452	9378
6-2FE	30281	6238	6048	4870	4870	4870	4811
7-1FE	75042	5396	5396	5070	5070	5070	5017
7-2FE	64473	5594	5594	5391	5391	5391	5344
8-1FE	56106	11248	11248	10729	10729	10729	10654
8-2FE	71599	18924	18924	18878	18878	18878	18837
9-1FE	56840	5744	5744	5029	5029	5029	4971
9-2FE	44798	4745	4745	3866	3866	3866	3775
17-1	93758	19667	19319	659	659	640	527
17-2	92512	15323	15106	393	393	359	309
17-3	103238	24010	23616	747	747	711	615
18-1	106526	27424	27062	979	979	972	895
18-2	110939	25164	24906	897	897	877	700
18-3	109956	27022	26592	983	983	948	799
19-1	104520	24265	23939	1097	1097	982	862

19-2	92454	13938	13709	529	529	447	374
19-3	95926	19374	19028	760	760	735	623
20-1	53646	5332	5222	106	106	106	103
20-2	108652	24276	23852	632	632	577	480
20-3	105363	21873	21427	617	617	476	404
Total	2865667	559389	555069	306621	306621	305935	302661
Percent Lost	N/A	80.48%	0.77%	44.76%	0.00%	0.22%	1.07%
Percent of Raw	100%	19.52%	19.37%	10.70%	10.70%	10.68%	10.56%

Supplementary Table 2. Total and named (does not have uncultured, unknown, etc… as part of name or is a repeat of the name of at the previous taxonomic level) taxa at each taxonomic level for digesta samples.

**Taxonomic Level**	**Total**	**Named**
Phylum	12	12
Class	18	24
Order	51	49
Family	81	70
Genus	142	108
Species	186	18

Supplementary Table 3. Permutational analysis of variance results.

Groups	t	Permutational p value	Unique permutations	Monte Carlo p value
50% DMM, 100% DMM	1.1577	0.022	933	0.205
50% DMM, 100% FM	1.1777	0.002	919	0.173
50% DMM, 50% WMM	1.3627	0.001	918	0.049
100% DMM, 100% FM	1.29	0.003	927	0.073
100% DMM, 50% WMM	1.3088	0.001	919	0.064
100% FM, 50% WMM	1.3899	0.001	921	0.025
