# Supplementary figures and images for: Effects of Fishmeal Substitution with Mealworm Meals (Tenebrio molitor and Alphitobius diaperinus) on the Growth, Physiobiochemical Response, Digesta Microbiome, and Immune Genes Expression of Atlantic Salmon (Salmo salar)
Source: Aquac Nutr. 2024 Jan 6;2024:6618117. doi: 10.1155/2024/6618117 (PMC10787657; doi:10.1155/2024/6618117)

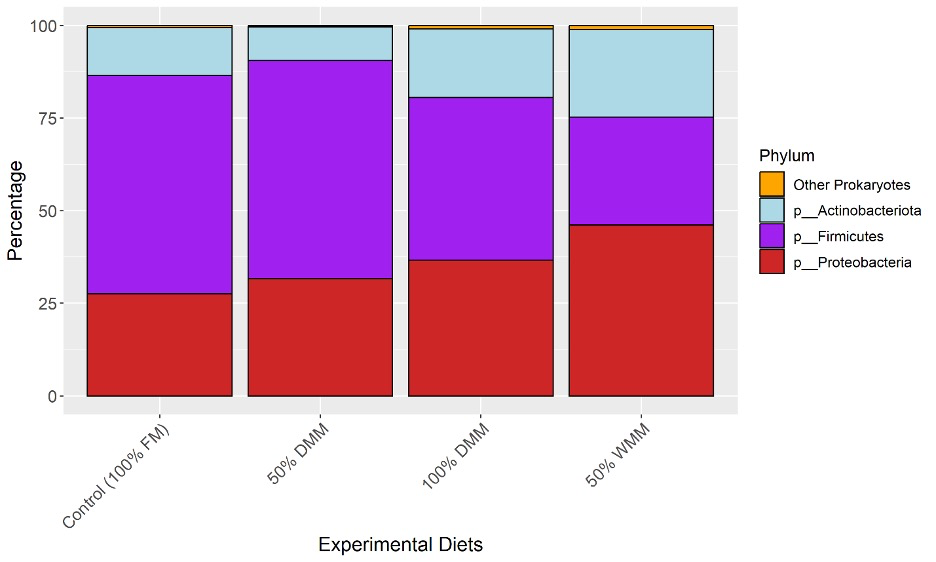

Supplement: Supplementary 2 — Figure S1: stacked bar graphs showing the phyla, which are represented by color, that had a mean relative abundance greater than 1% across all samples summarized by experimental treatment. [file 6618117.f2.png]
